# Severe Fever with Thrombocytopenia Syndrome Complicated by Co-infection with Spotted Fever Group Rickettsiae, China

**DOI:** 10.3201/eid2211.161021

**Published:** 2016-11

**Authors:** Qing-Bin Lu, Hao Li, Pan-He Zhang, Ning Cui, Zhen-Dong Yang, Ya-Di Fan, Xiao-Ming Cui, Jian-Gong Hu, Chen-Tao Guo, Xiao-Ai Zhang, Wei Liu, Wu-Chun Cao

**Affiliations:** Peking University School of Public Health, Beijing, China (Q.-B. Lu);; State Key Laboratory of Pathogen and Biosecurity, Beijing Institute of Microbiology and Epidemiology, Beijing (H. Li, P.-H. Zhang, Y.-D. Fan, X.-M. Cui, J.-G. Hu, C.-T. Guo, X.-A. Zhang, W. Liu, W.-C. Cao);; 154 Hospital of People's Liberation Army, Xinyang, China (N. Cui, Z.-D. Yang)

**Keywords:** spotted fever group rickettsiae, SFGR, severe fever with thrombocytopenia syndrome, SFTS, severe fever with thrombocytopenia syndrome virus, SFTSV, viruses, bacteria, zoonoses, epidemiologic characteristics, clinical features, laboratory findings, co-infection, complications, ticks, tick bites, tick-borne pathogen, human infection, China, vector-borne infections

## Abstract

During 2013–2015 in central China, co-infection with spotted fever group rickettsiae was identified in 77 of 823 patients infected with severe fever with thrombocytopenia syndrome virus. Co-infection resulted in delayed recovery and increased risk for death, prompting clinical practices in the region to consider co-infection in patients with severe fever with thrombocytopenia syndrome.

In recent years, new tickborne pathogens have increasingly emerged, creating public health challenges. Co-infection may occur in humans either through the bite of 1 tick co-infected with multiple pathogens or bites of multiple ticks, each carrying a different pathogen (*1*).

In 2009, severe fever with thrombocytopenia syndrome virus (SFTSV) was identified in humans in China, and since then, the virus has been detected in 19 provinces (*2*). The most highly affected region is in central China, where over one third of cases have been reported. Another tickborne pathogen, *Candidatus* Rickettsia tarasevichiae, classified among the spotted fever group rickettsiae (SFGR), was first identified in 2012 in the northeastern area of China, but is now infecting humans in the more densely populated central region (*3*). SFGRs have been detected in *Haemaphysalis longicornis* ticks (*3*,*4*), which also serve as a competent vector for SFTSV (*5*). In 2014, *Candidatus* R. tarasevichiae infection was detected in SFTSV-infected persons in eastern central China, indicating that co-infection with SFGR might be common among SFTSV-infected persons in the region (*3*). To determine the effects of co-infection with SFGR in SFTSV-infected persons, we compared clinical characteristics and laboratory findings for patients with SFTSV infection only with those for patients co-infected with SFTSV and *Candidatus* R. tarasevichiae.

## The Study

During 2013–2015, we conducted a retrospective investigation at the 154 Hospital of the People’s Liberation Army in Xinyang City, Henan Province, China. All patients meeting the criteria for having suspected severe fever with thrombocytopenia syndrome (SFTS) were enrolled (*6*). Serial serum and anti-coagulated blood samples were collected from patients throughout hospitalization and during convalescence.

RNA detection by reverse transcription PCR and serologic testing by ELISA were performed for diagnosis of SFTSV infection (*6*). SFTSV infection was determined by the detection of viral RNA in serum, seroconversion, or a 4-fold increase in SFTSV-specific IgG titers in paired serum samples collected >2 weeks apart. We used an indirect immunofluorescence assay (Focus Diagnostic, Cypress, CA, USA) to detect *Rickettsia rickettsii* IgG. Acute SFGR infection was defined as seroconversion or a 4-fold increase in *R. rickettsii* IgG titers in paired serum samples. We measured serum levels of cytokines and chemokines by using a Bio-Plex Pro Human Cytokine 27-plex Assay (Bio-Rad, Hercules, CA, USA).

For the study, we recruited 823 SFTS patients who had paired serum samples available for testing (Technical Appendix Table 1). Of those patients, 77 (8.5%) also had serologic evidence of SFGR infection: 45 showed seroconversion, and 32 had a 4-fold increase in IgG titers. Those 77 patients represented the SFTSV–SFGR co-infection group (Technical Appendix Table 2); the other 746 patients represented the SFTSV single-infection group.

Influenza-like symptoms were the most common clinical manifestations in both groups, and, except for fever, which was more prolonged in the co-infection group (p = 0.039), symptoms were comparable in the groups (Technical Appendix Table 3). Ascites and hemorrhagic signs were more common in the co-infection than the single-infection group (p = 0.002 and p = 0.003, respectively). The frequencies of other complications, including gastrointestinal, respiratory, and neurologic syndromes, were similar in the 2 groups.

**Figure 1:**
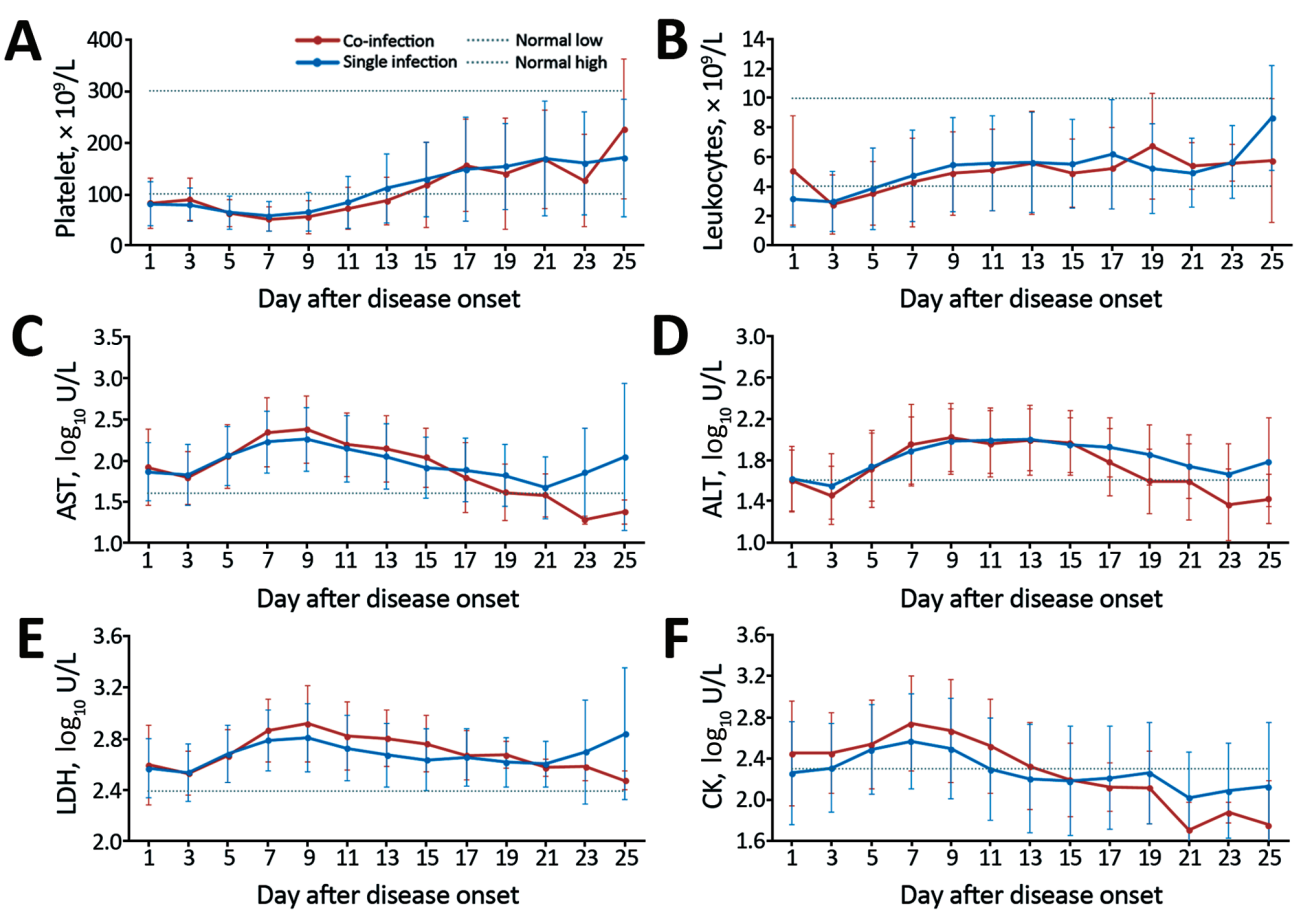
Dynamic profiles for 6 selected laboratory parameters for hospitalized patients with severe fever with thrombocytopenia syndrome virus (SFTSV) infection only or with SFTSV and spotted fever group rickettsiae co-infection, China, 2013–2015. A–B) Mean counts and 95% CIs (error bars) for platelets (A) and leukocytes (B). C–F) log_10_-transformed median level of and interquartile ranges (error bars) for aspartate aminotransferase (AST) (C); alanine aminotransferase (ALT) (D); lactate dehydrogenase (LDH) (E); and creatine kinase (CK) (F). Dashed lines indicate the reference level for each parameter. Parameters were compared by using the generalized estimating equation model.

At hospital admission, the co-infection group had longer prothrombin times (Table). For both groups, thrombocytopenia occurred starting at 4 days after symptom onset and persisted for as long as 2 weeks (Figure 1). Using log_10_-transformed data with the generalized estimating equation model, we showed that platelet count and leukopenia recovery were delayed in the co-infection group compared with the single-infection group (p = 0.045 and p = 0.027, respectively). The generalized estimating equation model also showed that the co-infection group had higher levels of serum creatine kinase (p = 0.047) and lactate dehydrogenase (p = 0.022) during those recovery processes.

**Table:** Laboratory test results for patients with severe fever with thrombocytopenia syndrome with and without co-infection with spotted fever group rickettsiae

Characteristics	Single infection, n = 77	Co-infection, n = 746	p value
Laboratory parameters on admission, no. (%) patients			
Leukocyte count <4 × 10^9^/L	60 (77.9)	613 (82.2)	0.358
Platelet count <100 × 10^9^/L	64 (83.1)	624 (83.7)	0.905
Neutrophils >70%	36 (46.8)	348 (46.7)	0.986
Lymphocytes <20%	27 (35.1)	276 (37.0)	0.738
Hemoglobin <110 g/L	9 (11.7)	113 (15.2)	0.416
Aspartate aminotransferase >40 U/L	62 (80.5)	621 (83.4)	0.527
Alanine aminotransferase >40 U/L	40 (52.0)	421 (56.6)	0.435
Albumin <35 g/L	10 (13.0)	90 (12.1)	0.817
Alkaline phosphatase >150 U/L	3 (3.9)	48 (6.4)	0.378
Gamma-glutamyl transpeptidase >50 U/L	22 (28.6)	161 (21.6)	0.164
Lactate dehydrogenase >245 U/L	60 (85.7)	599 (83.9)	0.691
Creatine kinase >200 U/L	49 (63.6)	465 (62.3)	0.822
Blood urea nitrogen >7.14 mmol/L	22 (28.6)	244 (32.9)	0.442
Total bilirubin >17.1 μmol/L	10 (13.0)	77 (10.3)	0.469
Ceatinine >97 μmol/L	14 (20.0)	130 (18.2)	0.716
Serum amylase >115 U/L	30 (56.6)	242 (52.4)	0.560
Calcium <2.1 mmol/L	34 (61.8)	389 (61.8)	0.997

Coagulation parameters, median (interquartile range)			
Prothrombin time, s	11.5 (10.4–12.3)	10.9 (10.2–11.6)	0.017
Thrombin time, s	20.6 (17.4–22.7)	19.7 (17.6–22.4)	0.318
Activated partial thromboplastin time, s	45.5 (40.5–58.5)	46.2 (38.8–56.6)	0.797
Fibrinogen, g/L	2.8 (2.2–3.2)	2.7 (2.3–3.2)	0.775
International normalized ratio	1.0 (0.9–1.1)	1.0 (0.9–1.0)	0.136
Prothrombin time activity, %	84.0 (76.2–92.3)	87.7 (79.7–98.1)	0.124
D-dimer, ng/mL	1,014 (356–1,432)	647 (381–1,373)	0.603

**Figure 2:**
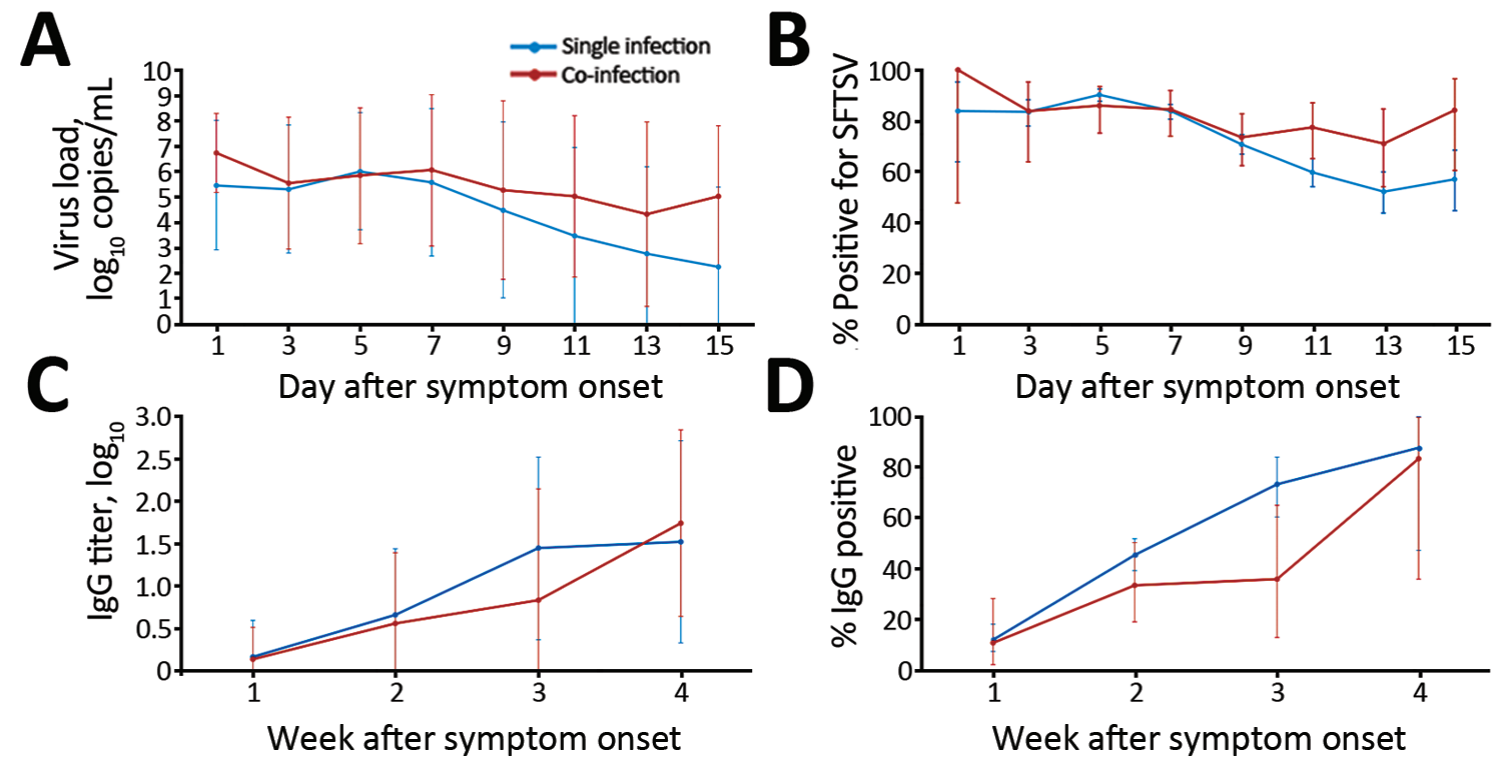
Dynamic profiles for severe fever with thrombocytopenia syndrome virus (SFTSV) RNA and SFTSV-specific IgG in hospitalized patients with SFTSV infection only or with SFTSV and spotted fever group rickettsiae co-infection, China, 2013–2015. A) log_10_-transformed SFTSV virus loads. B) Percentage of patients positive for SFTSV. C) log_10_-transformed SFTSV IgG titers. D) Percentage of patients positive for SFTSV IgG. Comparisons were performed using the generalized estimating equation model. The error bars, which show the standard deviation for log_10_-transformed SFTSV virus loads and log_10_-transformed SFTSV IgG titers, represent the 95% CI for the percentage of patients positive for SFTSV and SFTSV IgG.

Based on the dynamic patterns at 2-day intervals, virus loads in the single-infection group peaked at day 5 after symptom onset and gradually decreased thereafter. Virus loads in the co-infection group peaked at day 7 and then deceased at a lower rate than that for the single-infection group after we adjusted for sex, age, and time from symptom onset to hospital admission (p = 0.028) (Figure 2, panel A).

At weeks 1 and 2 after symptom onset, SFTSV-specific IgG titers and positivity rates were not significantly different between the 2 groups (Figure 2, panels C, D). At week 3, the co-infection group had a significantly lower rate of SFTSV positivity (p = 0.007). Antibody titers at week 4 were not significantly different between the groups (Figure 2, panel C).

We conducted laboratory testing for 34 patients with SFTSV–SFGR co-infection, 30 sex- and age-matched patients with SFTS only, and 25 controls who were negative for both pathogens by molecular and antibody testing. Levels of interleukin (IL)–1 receptor agonist, IL-8–10, IL-17, interferon-γ, monocyte chemoattractant protein 1, monocyte chemoattractant protein α1, granulocyte colony-stimulating factor, fibroblast growth factors, and tumor necrosis factor–α were similar in the single-infection and co-infection groups and significantly elevated compared with levels in the control group (online Technical Appendix Figure). IL-6 and IL-15 levels were elevated in both infection groups, but they were significantly higher in the SFTSV single-infection group. Platelet-derived growth factor–BB and RANTES (regulated on activation, normal T cell expressed and secreted) were decreased in both groups, but we observed intergroup differences only for RANTES.

Altogether, 87 (10.6%) patients died. The case-fatality rate in the co-infection group (16.9% [13/77]) was insignificantly higher than that in the single-infection group (9.9% [74/746]) (p = 0.058). The association between co-infection and higher case-fatality rate was significant after adjustment for sex, age, and interval from disease onset to hospital admission (odds ratio 1.992, 95% CI 1.025–3.873; p = 0.042) (Technical Appendix Table 4).

## Conclusions

Our retrospective investigation in an SFTSV-endemic region of China identified SFTSV–SFGR co-infection in ≈8.5% of SFTSV-infected patients and a higher frequency of fatal outcome and delayed recuperation in the co-infected patients. These findings highlight the importance of considering SFGR infection in the differential diagnosis for patients in SFTSV-endemic regions.

SFTSV infection can cause a wide variety of signs and symptoms, ranging from influenza-like illness to more severe complications and even life-threatening disease (*7*). Complications usually involve neurologic and hemorrhagic manifestations, which can progress to multiple organ dysfunction in critically ill patients. Rickettsial infections are clinically difficult to distinguish from many virus infections (*8*), and our results showed that symptoms common to SFTSV- and SFGR-infected patients (e.g., influenza-like illness, gastrointestinal symptoms) are not intensified in co-infected patients. In contrast, less common hemorrhagic signs, especially gastrointestinal hemorrhages, are exacerbated in co-infected patients. Previous studies have shown that in patients with SFTS, blood coagulation parameters are prolonged, as characterized by activated partial thromboplastin time and thrombin time (*9*,*10*). Thrombocytopenia, a common laboratory finding in patients with SFTS, can contribute to hemorrhage, and hemorrhagic signs have also been observed in patients infected with SFGR species (e.g., *R. rickettsii* and *R. conorii*) (*12*–*14*); however, SFGR mainly invade the vascular endothelial cells, causing vascular inflammation and increased vascular permeability (*11*). Also, based on prolonged thrombocytopenia and longer prothrombin times that have been observed in co-infected persons, we hypothesize that the additive effect from 2 pathogens might lead to aggravated hemorrhage.

Doxycycline is the recommended therapeutic regimen for rickettsia infection (*15*) and could be administered in cases of SFTSV–SFGR co-infection. From a public health perspective, intensified efforts should be made to detect SFTSV–SFGR co-infection in regions where *H. longicornis* ticks predominate and carry both SFTSV and SFGR.

Technical Appendix Detailed study methods and characteristics of patients with severe fever with thrombocytopenia syndrome virus (SFTSV) infection only or with SFTSV and spotted fever group rickettsiae co-infection.
