# Supplementary material for: Severe Fever with Thrombocytopenia Syndrome Complicated by Co-infection with Spotted Fever Group Rickettsiae, China
Source: Emerg Infect Dis. 2016 Nov;22(11):1957–60. doi: 10.3201/eid2211.161021 (PMC5088031; doi:10.3201/eid2211.161021)
Supplement: Technical Appendix — Detailed study methods and characteristics of patients with severe fever with thrombocytopenia syndrome virus (SFTSV) infection only or with SFTSV and spotted fever group rickettsiae co-infection. [file 16-1021-Techapp-s1.pdf]

Article DOI: http://dx.doi.org/10.3201/eid2211.161021

Severe Fever with Thrombocytopenia Syndrome Complicated by Co-infection with Spotted Fever Group Rickettsiae, China

Technical Appendix

Methods

SFTSV Detection and Quantitation by Real-time PCR

Serial anti-coagulated blood and serum samples were collected from clinically suspected patients, at entry into the hospital and during their hospitalization. Viral RNA was isolated from serum samples using QIAamp Viral RNA Mini Kit (Qiagen, Germantown, MD, USA), according to the manufacturer's instructions. One-step Primer Script RT-PCR Kit (TaKaRa Bio dalian Inc., Japan) was used according to the manufacturer's instructions for SFTSV detection in a volume of 20 µL containing 10 µL of One Step RT-PCR Buffer (2×), 0.4 µL of TaKaRa Ex Taq HS (5 U/µL) and 0.4 µL of PrimeScript RT Enzyme MixII (TaKaRa Bio dalian Inc., Japan), 1 µL of PCR primer mix (20 µM of sense and antisense each) and 0.5µL of Probe (10 µM), total RNA 2 µL and RNase free dH_2O (5.7 µL). PCR was carried with one cycle of 42°C for 5 min and 95°C for 10 s, followed by 40 cycles of 95°C for 5 s and 55°C for 20 s in a LightCycler Real Time PCR apparatus (Roche Diagnostics, Mannheim, Germany). The real-time PCR primers and probe were targeted at the S-segment of the SFTSV. The sequences were as follows: forward:TTCACAGCAGCATGGAGAGG; reverse: GATGCCTTCACCAAGACTATCAATG;

Probe: AACTTCTGTCTTGCTGGCTCCGC. Nested RT-PCR and sequencing of the M-segment were performed on randomly selected positive samples to verify the real-time RT-PCR

results. We have adopted several strategies to prevent contamination. All negative extraction controls were included to detect cross-contamination events within each extraction. To rule out contamination of the PCR reagents or cross-contamination of the PCR plate, water was included as a control, and RNA from the original specimens was capped before the addition of any positive control materials.

Quantitation of virus was performed using quantitative RT-PCR targeting the same gene segments. Real-time PCR was performed in a volume of 20 μL by use of the One-step Primer Script RT-PCR Kit (TaKaRa Bio dalian Inc., Japan) in the CFX96 instrument (Bio-Rad, California, USA). Standard curves or absolute RNA quantification were included in every assay and were generated by using RNA transcripts produced by in vitro transcription of cDNA that included the real time qPCR assay amplicons. Standard curves included five dilutions and three replicate wells for each dilution. All samples were quantified in at least duplicate wells. Levels of SFTSV RNA concentrations were expressed as copies/mL.

SFTSV-specific IgG antibody detection by ELISA

SFTSV-specific IgG antibodies were detected by enzyme-linked immunosorbent assay (ELISA) using the same protocol described previously (*1*). Briefly, 96-well polystyrene microtiter plates were coated with purified SFTSV antigen. Serially diluted serum samples starting with 1:10 were added, and after incubation and washing, antihuman IgG conjugates were added. Substrate solution was added for color development. The optical density at 450 nm (A450) of each well was measured. The cutoff limit was 0.1 plus the optical density of the negative control, according to the manufacturer’s instructions.

Determination of serum cytokine/chemokine concentration

Serum levels of 27 cytokines and chemokines were measured with the use of Bio-plex Pro Human 27-plex cytokine panel (Bio-plex Pro Human 27-plex cytokine panel, Bio-Rad, CA, USA). The tested cytokines included: interleukin (IL)-1β, IL-1 receptor antagonist (IL-1RA), IL-2, IL-4,

IL-5, IL-6, IL-7, IL-8, IL-9, IL-10, IL-12, IL-13, IL-15, IL-17, eotaxin, granulocyte colony-stimulating factor (G-CSF), granulocyte macrophage colony-stimulating factor (GM-CSF), basic FGF, interferon (IFN)-γ, IFN-γ-inducible protein (IP-10), monocyte chemotactic protein-1 (MCP-1), macrophage inflammatory protein 1a (MIP-1α), macrophage inflammatory protein 1β (MIP-1β), platelet-derived growth factor (PDGF-BB), regulated on activation and normally T-cell expressed (RANTES), tumor necrosis factor (TNF)-α, and vascular endothelial growth factor (VEGF). Following manufacturer's instruction, a multiplex-biometric immunoassay containing fluorescent microspheres conjugating with monoclonal antibodies specific for target cytokines was performed to test serum cytokine levels. Cytokine concentrations were calculated from standard curves of known concentrations of recombinant human cytokines. Levels were analyzed using Bio-Plex Manager 6.0 software.

Data Collection

We performed a chart review of all recruited patients, collected information on demographic characteristics, symptoms and signs, clinical laboratory test results and treatment regimens, etc. These data were drawn from the medical database by a group of trained physicians using a standardized format and entered into an EpiData database. The data were further reviewed for accuracy and consistency by a group of epidemiologists.

Main Outcome Measurements

The main outcome measurements, including hemorrhagic manifestations, presence of plasma leakage, neurologic manifestations, respiratory manifestations and death, were consecutively reviewed during the whole hospitalization. The hemorrhage manifestations were defined by presence of petechiae, ecchymosis, hemoptysis, epistaxis, hematuria, hematemesis, melena, vaginal, and gingival bleeding. Plasma leakage was determined by the presence of pleural and/or ascitic fluid or haemoconcentration. Deaths were classified based on clinical diagnoses of the proximate cause of death by the physician.

Statistical Analyses

Categorical variables were assessed using the χ^2 test or the Fisher exact test. Continuous variables were compared by Student's t-test and assessed with the nonparametric Mann-Whitney U test in case of skewed variables. The comparisons of laboratory parameters every 2 days from disease onset to discharge were performed by generalized estimating equation (GEE) with adjustment for the variables of age, sex and the intervals from disease onset to hospital admission. All analyses were performed by SAS software, version 9.1.3 (SAS Institute, Cary, North Carolina, USA). All p values were 2-tailed and $p<0.05$ was considered statistically significant.

Results

In total 1080 laboratory-confirmed SFTS patients were hospitalized during the study period, among them 823 patients who had convalescence serum samples collected were recruited into the study. Of the 823 patients, 25 (3.0%) patients were negative for SFTSV RT-PCR and had seroconversion (n = 18) or a 4-fold increase (n = 7) in titers of IgG antibody (Technical Appendix Table 1). Other 798 patients were positive for SFTSV RT-PCR assay. Seventy-seven (8.5%) of the 823 patients provided serologic evidence of SFGR infection according to seroconversion (n = 45) or 4-fold increase of antibody titer (n = 32), composing as the SFTSV-SFGR coinfection group (Technical Appendix Table 2).

Reference

1. Yu XJ, Liang MF, Zhang SY, Liu Y, Li JD, Sun YL, et al. Fever with thrombocytopenia associated with a novel bunyavirus in China. N Engl J Med. 2011;364:1523–32. PubMed http://dx.doi.org/10.1056/NEJMoa1010095

Technical Appendix Table 1. The titers of IgG antibody in the SFTS patients with RT-PCR negative result tested by ELISA

Patient No.	Titer of IgG antibody at acute stage	Titer of IgG antibody at recovery stage
1	1:10	1:1280
2	<1:10	1:640
3	<1:10	1:640
4	<1:10	1:10
5	1:20	1:160
6	<1:10	1:640
7	<1:10	1:80
8	<1:10	1:20
9	<1:10	1:20
10	<1:10	1:10
11	<1:10	1:10
12	1:10	1:40
13	<1:10	1:10
14	<1:10	1:80
15	<1:10	1:20
16	1:10	1:80
17	<1:10	1:10
18	1:80	1:320
19	1:10	1:320
20	1:10	1:320
21	<1:10	1:40
22	<1:10	1:10
23	<1:10	1:160
24	<1:10	1:10
25	<1:10	1:20

Technical Appendix Table 2. The titers of IgG antibody for spotted fever group rickettsiae tested by IFA

Patient No.	Titer of IgG antibody at acute stage	Titer of IgG antibody at recovery stage
3	<1:64	1:256
4	<1:64	1:128
9	1:64	1:512
16	<1:64	1:512
18	<1:64	1:256
26	<1:64	1:64
27	<1:64	1:64
28	<1:64	1:64
29	<1:64	1:64
30	<1:64	1:64
31	1:64	1:256
32	1:64	1:256
33	<1:64	1:64
34	<1:64	1:128
35	<1:64	1:128
36	<1:64	1:128
37	<1:64	1:128
38	<1:64	1:128
39	<1:64	1:128
40	<1:64	1:128
41	<1:64	1:128
42	<1:64	1:128
43	<1:64	1:128
44	<1:64	1:256
45	<1:64	1:256
46	<1:64	1:256
47	<1:64	1:256
48	<1:64	1:128
49	<1:64	1:128
50	<1:64	1:512
51	<1:64	1:512
52	<1:64	1:128
53	1:64	1:512
54	1:64	1:256
55	1:64	1:256
56	1:64	1:256

Patient No.	Titer of IgG antibody at acute stage	Titer of IgG antibody at recovery stage
57	1:64	1:512
58	1:64	1:512
59	1:64	1:256
60	1:64	1:512
61	1:64	1:256
62	1:64	1:1024
63	1:64	1:256
64	1:64	1:512
65	1:64	1:256
66	1:64	1:512
67	1:64	1:1024
68	1:64	1:512
69	1:64	1:512
70	<1:64	1:512
71	<1:64	1:256
72	<1:64	1:256
73	<1:64	1:256
74	<1:64	1:256
75	<1:64	1:256
76	<1:64	1:256
77	1:64	1:256
78	1:64	1:256
79	1:64	1:256
80	<1:64	1:128
81	<1:64	1:128
82	<1:64	1:128
83	<1:64	1:128
84	<1:64	1:128
85	<1:64	1:128
86	<1:64	1:128
87	<1:64	1:128
88	<1:64	1:128
89	<1:64	1:64
90	<1:64	1:64
91	<1:64	1:64
92	<1:64	1:64
93	<1:64	1:64
94	<1:64	1:64

Patient No.	Titer of IgG antibody at acute stage	Titer of IgG antibody at recovery stage
95	<1:64	1:64
96	<1:64	1:64
97	<1:64	1:64

Technical Appendix Table 3. Demographic and clinical characteristics of the patients with SFTSV-SFG rickettsiae co-infection *vs.* SFTSV single infection

Characteristics	Co-infection (n = 77)	Single infection (n = 746)	*P* value
Demographic characteristics			
Age, mean ± SD, years	61.4 ± 12.6	61.8 ± 12.2	0.809
Sex, female, n (%)	42 (54.6)	442 (59.3)	0.425
Hospital duration, median (IQR)	8(5–10)	7 (5–9)	0.137
Delay days from onset to admission, median (IQR)	5 (4–7)	5 (4–7)	0.917
Days>5 from disease onset, n (%)	38 (49.4)	355 (47.6)	0.768
Tick bite history, n (%)	10 (13.0)	87 (11.7)	0.731
Underlying diseases, n (%)	32 (41.6)	244 (32.7)	0.016
Cardiovascular and cerebrovascular diseases	11 (14.3)	75 (10.1)	0.248
Diabetes	18 (23.4)	144 (19.3)	0.392
Active hepatitis	7 (9.1)	46 (6.2)	0.320
Chronic respiratory disease	2 (2.6)	14 (1.9)	0.663
Clinical manifestations			
Influenza like illness	77 (100)	746 (100)	1.000
Febrile, n (%)	76 (98.7)	744 (99.7)	0.256
Fever duration, days, median (IQR)	7 (5–9)	6 (4–8)	0.039
Lymphadenectasis, n (%)	33 (42.9)	334 (44.8)	0.748
Myalgia, n (%)	57 (74.0)	608 (81.5)	0.113
Weakness, n (%)	73 (94.8)	722 (96.8)	0.362
Dizziness, n (%)	16 (20.8)	121 (16.2)	0.307
Headache, n (%)	10 (13.0)	54 (7.2)	0.073
Chills, n (%)	7 (9.1)	60 (8.0)	0.749
Arthralgia, n (%)	2 (2.6)	10 (1.3)	0.381
Gastrointestinal illness, n (%)	56(96.1)	509 (68.2)	0.418
Abdominal pain	3 (3.9)	25 (3.4)	0.802
Nausea	53 (68.8)	469 (62.9)	0.398
Diarrhea	20 (26.0)	146 (19.6)	0.183
Vomit	24 (35.2)	196 (26.3)	0.848
Diarrhea and Vomit	36 (46.8)	288 (38.6)	0.164
Plasma leakage, n (%)	9 (11.7)	70 (9.4)	0.513
Hydrothorax	8 (10.4)	64 (8.6)	0.592

Characteristics	Co-infection (n = 77)	Single infection (n = 746)	*P* value
Hydropericardium	1 (1.3)	12 (1.6)	0.836
Ascites	3 (3.9)	4 (0.5)	0.002
Respiratory syndrome, n (%)	34 (44.2)	377 (50.5)	0.286
Dyspnea	5 (6.5)	4 (2.7)	0.172
Sputum	22 (28.6)	264 (35.4)	0.232
Cough	33 (42.9)	355 (47.6)	0.429
Hemorrhage manifestations, n (%)	38 (49.4)	244 (32.7)	0.003
Melena	8 (10.4)	34 (4.6)	0.027
Gingival bleeding	9 (11.7)	60 (8.0)	0.272
Hemoptysis	2 (2.6)	15 (2.0)	0.730
Haematemesis	2 (2.6)	29 (3.9)	0.571
Hematuria	13 (16.9)	63 (8.5)	0.074
Petechial/ecchymosis	20 (26.0)	119 (16.0)	0.025
Epistaxis	0 (0)	7 (0.9)	0.393
Neurologic symptom, n (%)	29 (37.7)	243 (32.6)	0.366
Coma	1 (1.3)	50 (6.7)	0.061
Dysphoria	11 (14.3)	71 (9.5)	0.184
Lethargy	1 (1.3)	40 (5.4)	0.690
Blurred mind	13 (16.9)	133 (17.8)	0.836
Convulsion	11 (14.3)	99 (13.3)	0.803
Apathy	15 (19.5)	143 (19.2)	0.947

Technical Appendix Table 4. The association between death and the related variables

	Univariate		Multivariate	
Variables	OR (95% CI)	*P*	OR (95% CI)	*P*
Model 1 in all the patients				
Group (coinfection/single)	1.845 (0.970–3.508)	0.062	1.992 (1.025–3.873)	0.042
Age	1.061 (1.036–1.087)	<0.001	1.061 (1.036–1.867)	<0.001
Sex (male/female)	1.882 (1.203–2.945)	0.006	1.716 (1.082–2.720)	0.022
Interval from onset to admission	1.142 (1.055–1.235)	0.001	1.107 (1.020–1.202)	0.015
Model 2 in the coinfection group				
Age	1.082 (1.014–1.155)	0.017	1.109 (1.023–1.202)	0.012
Sex (male/female)	9.167 (1.870–44.922)	0.006	12.491 (1.977–78.910)	0.007
Interval from onset to admission	0.757 (0.160–4.963)	0.895	0.860 (0.585–1.265)	0.443
Model 3 in SFTSV single group				
Age	1.063 (1.037–1.090)	<0.001	1.057 (1.030–1.084)	<0.001
Sex (male/female)	1.517 (0.938–2.454)	0.090	1.314 (0.799–2.160)	0.282
Interval from onset to admission	1.182 (1.086–1.286)	<0.001	1.142 (1.047–1.247)	0.003

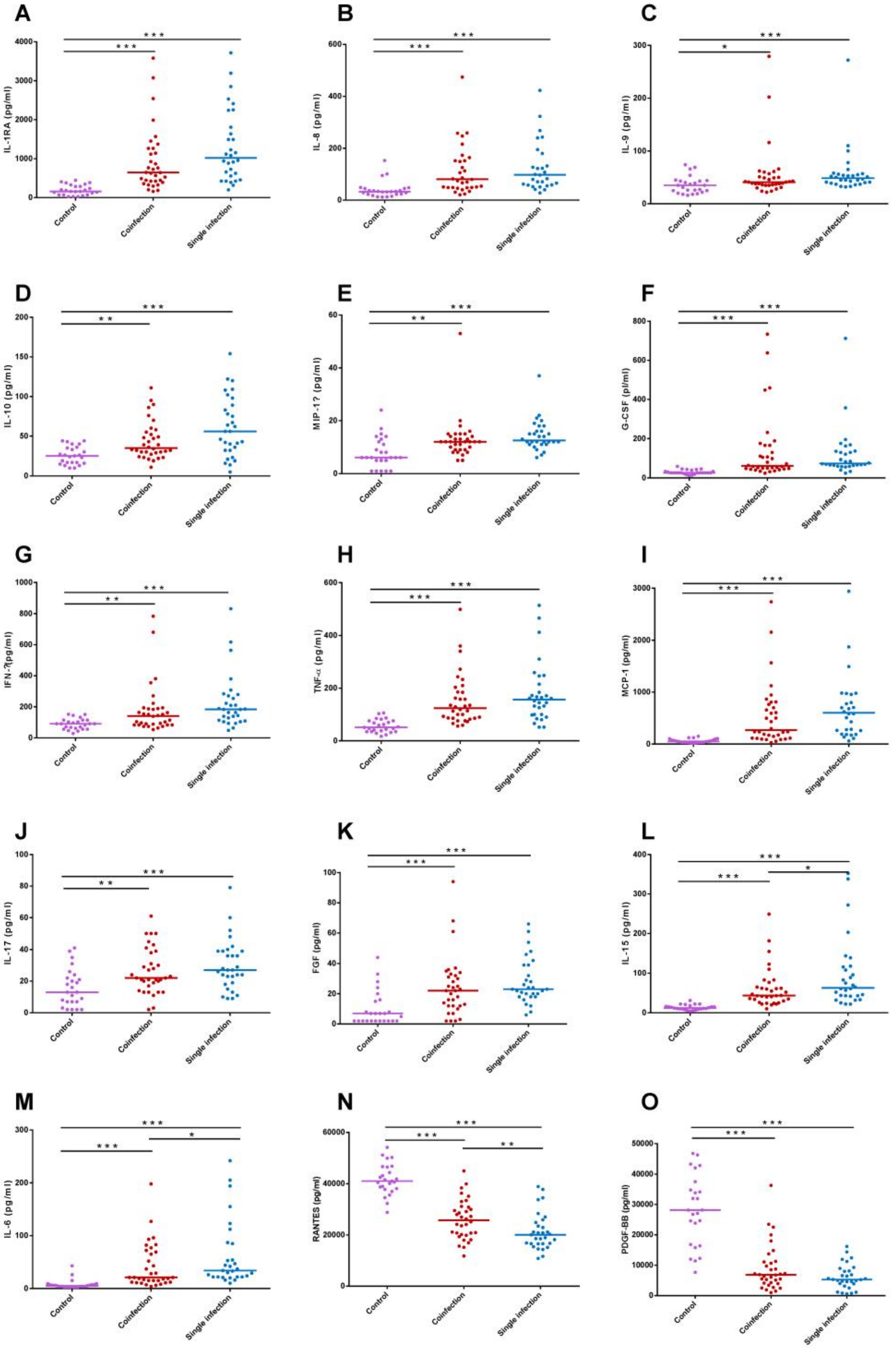

Technical Appendix Figure. Comparison of cytokine production in two groups of patients. *p<0 0.05, **p<0 0.01, ***p<0 0.001. Altogether 34 coinfected patients, 30 age and gender comparable SFTS patients and 25 healthy controls who showed no positive SFTSV or *R. rickettsii* detection by either molecular or antibody tests were measured. The 27 cytokines were identified with different patterns of production.

Cytokines IL1-RA, IL-8, IL-9, IL-10, IFN-γ, MCP-1α, G-CSF, FGF, TNF-α, MCP-1 and IL-17 were significantly elevated in two groups than controls, yet showing no inter-group difference (Figure, panels A–K). Cytokine IL-6 and IL-15 were elevated in both groups, with SFTSV single infection attaining significantly higher level than the coinfection group (Figure, panels L, M). PDGF-BB and RANTES was decreased in both groups (Figure, panels N, O), and showing inter-group difference for RANTES only (Figure, panel N). The remaining cytokines were within the normal level and displayed no significant difference between two groups. Abbreviations: G-CSF, granulocyte colony-stimulating factor; IL, interleukin; RANTES, regulated on activation and normally T-cell expressed. PDGF: platelet-derived growth factor.
